# The Role of Territorial Planning Policies in Groundwater Recharge

**DOI:** 10.1007/s00267-026-02642-7

**Published:** 2026-11-06

**Authors:** Fabiane Wiederkehr, Clódis de Oliveira Andrades-Filho, Ana Maria Pimentel Mizusaki

**Affiliations:** 1 Instituto de Geociências, Programa de Pós-Graduação em Geociências, Universidade Federal do Rio Grande do Sul, Campus do Vale, Av. Bento Gonçalves, 9500, Porto Alegre, 91501-970 Brazil; 2 Instituto de Geociências, Programa de Pós Graduação em Sensoriamento Remoto, Universidade Federal do Rio Grande do Sul, Campus do Vale, Av. Bento Gonçalves, 9500, Porto Alegre, 91501-970 Brazil; 3 Universidade Estadual do Rio Grande do Sul, Uergs Hortênsias, Rua Assis Brasil, 842, São Francisco de Paula, 95400-000 Brazil

**Keywords:** Land-use policy, Disaster management, Water security, Volcanic plateau, Grasslands

## Abstract

Identifying and preserving groundwater recharge potential zones (GWRPZs) is essential for integrating water security into territorial planning. While maintaining high recharge potential theoretically supports subsurface water storage and landscape resilience against extreme weather events, long-term spatial evaluations of how land-use policies preserve these functions remain limited. Despite the abundance of water resources in Brazil, insufficient coordination between land-use planning and groundwater management compromises water security. There is a knowledge gap regarding long-term spatial-temporal evaluations of how territorial policies, rather than land use alone, affect the maintenance of groundwater recharge potential. This study addresses this gap by assessing the impact of territorial policies on aquifer recharge potential over time. The purpose of this paper is to demonstrate the need for territorial policies governing freshwater management to support long-term water security. This study examined a volcanic plateau in southern Brazil to illustrate a framework. Using a GIS approach, this paper established a method for evaluating GWRPZ changes between 1985 and 2022 and compared these changes with territories under different land-use restrictions. The resulting GWRPZ change map showed areas where recharge potential had improved and others where it had been reduced between 1985 and 2022. Higher GWRPZ maintenance was associated with a higher degree of area protection. Agriculture was identified as the main pressure on GWRPZs, whereas the main resistance was forest recovery. These data can help inform policy decision-making to identify more applicable and appropriate solutions for sustainable development.

## Introduction

Water is essential for human health and economic development. The sixth Sustainable Development Goal (SDG) is to ensure everyone has access to safe water and sanitation. Beyond that, water quality and quantity are central to achieving many of the other SDGs (United Nations, 2024). Water is essential for sustainable development in its truest sense and full meaning of the word sustainability (Abu-Zeid, 1998).

Water management has faced many challenges, especially related to extreme weather events resulting from climate change. Even in countries with abundant freshwater resources, such as Brazil, scarcity and flooding have become increasingly frequent (AghaKouchak et al., 2020; Ávila et al., 2016; Cai et al., 2020; Gosling and Arnell, 2016; Marengo et al., 2023; Veldkamp et al., 2015).

Well-known freshwater challenges have been exacerbated by global warming and climatic fluctuations. These challenges include water scarcity, lack of access to clean drinking water and sanitation, water quality deterioration, fragmentation of water management, declines in financial resources, threats to world peace and security, and a lack of awareness by decision-makers and the public about the magnitude of the problem (Abu-Zeid, 1998). Finally, unsustainable land use increases vulnerability to disasters (Azadi et al., 2022).

The key pressures on drinking water quality are construction land development and farming, while forests and wetlands are the primary resistances (Wang et al., 2024). Ideally, drinking water protection should focus on raising source water quality rather than treatment sophistication, because treatment alone is not failsafe, but rather should be part of a multi-layered approach (Davies and Mazumder, 2003).

There is a great need for efficient water resource protection and management. The crisis is growing fast, and the usual water management strategies are no longer enough in the face of changes in the water cycle. Water security management requires effective policies that identify, document, and reduce watershed risks. It’s imperative to recognize the role of strong watershed management and the connection between policymakers, policy instrumentation, managers, and scientists (Davies and Mazumder, 2003).

The management of groundwater recharge zones has multiple benefits. Groundwater itself is the most significant and reliable freshwater resource globally (Abdo et al., 2024). Moreover, groundwater is connected to surface water and influences, as well as being influenced by, its quality and quantity (Doke et al., 2021). Infiltration in these zones theoretically functions as a natural mechanism for water retention. Higher spatial recharge potential can support subsurface water storage, which is widely recognized in hydrogeological literature as contributing to streamflow regulation during seasonal variations (Aloui et al., 2024). This regulatory role is especially important in the context of climate change, which is increasing the frequency and severity of extreme events (AghaKouchak et al., 2020). In headwater regions like the studied volcanic plateau, mapping and protecting high recharge potential zones is a key spatial planning strategy to identify natural infrastructure that supports regional water security.

Although groundwater levels have been rapidly declining in aquifers globally, there are some cases in which depletion trends have reversed. These were attributed to policy changes, managed aquifer recharge, and surface-water diversions (Jasechko et al., 2024). This demonstrates the potential for depleted aquifer systems to recover and for watersheds to re-equilibrate their water balance following efficient territorial planning policies.

The combination of remote sensing and geographic information systems (GIS) is an efficient technique to delineate large-scale groundwater recharge potential zones (GWRPZs) (Abdullateef et al., 2021). These GIS-based maps are a valuable tool for directing detailed site-specific studies and aiding decision-makers in sustainable groundwater management, optimized land use planning, and holistic development (Aloui et al., 2024). Such an approach is particularly vital in terrains of low-porosity crystalline and volcanic rocks, where water is stored predominantly in fractures.

A large portion of the world’s groundwater is stored in fractures because of the widespread presence of crystalline, low-porosity rocks across major continents. In many areas, volcanic rocks are the dominant rock type, and their groundwater is widely utilized because it is available throughout all seasons and is often of high quality and abundant (Baud et al., 2024), especially in continental flood basalt plateaus (Vaccaro, 1999). Fractured and weathered parts of volcanic rocks provide groundwater for both rural and urban areas in various countries all over the world.

In Ethiopia, for example, more than 90% of the freshwater used for domestic and industrial supply comes from groundwater, dominantly from volcanic aquifers (Fenta et al., 2020). In the United States of America, the Columbia River Volcanic Plateau has faced a long-term water level decline, which is concerning due to a general lack of knowledge about natural recharge (Piersol and Sprenke, 2015). In the Deccan Volcanic Province, the failure of boreholes is common due to overexploitation and a lack of adequate knowledge in site selection, which has pushed many of the farmers into a spiraling debt and ultimately to suicide (Thomas and Duraisamy, 2018). In Namibia, the Etendeka volcanic plateau has little hydrogeological potential and is highly dependent on the occurrence of rainfall (Christelis et al., 2001).

The knowledge gap identified in these global contexts highlights that, to ensure long-term water security, it is not enough to identify where recharge occurs; it is necessary to understand how territorial policies influence the maintenance of these zones over time. This study advances beyond traditional static models by proposing an innovative methodology that evaluates temporal changes in recharge potential between the 1980s and the 2020 s. The use of GIS is essential: it enables the synthesis of multiple determining factors, such as lineament density and slope gradient, through a multi-criteria decision analysis capable of evaluating how different land-use restriction regimes influence the spatial maintenance and loss of groundwater recharge potential zones over time.

To illustrate the framework, this study examined a volcanic plateau in southern Brazil, a critical headwater region that feeds three major drainage basins and supports more than half of the Rio Grande do Sul state’s population.

Therefore, the main contribution of this research lies in evaluating the spatial-temporal relationship between land-use planning policies and GWRPZ dynamics by comparing territories with varying levels of legal protection. By showing how different protection regimes are associated with the maintenance or loss of recharge potential, this study provides a spatial-temporal framework to guide territorial planning policies and identify priority areas where land-use regulation may help preserve natural recharge conditions.

## Protected Areas

In Brazil, land use restriction policies aim to protect the environment and ensure sustainable development.

Brazilian National Water Resources Policy was instituted by Law n. 9433 (Brasil, 1997). It defines the watershed as the territorial unit for planning and managing water issues. One of its objectives is coordination between water and land use management. It also ensures Water Resources Plans may propose the creation of areas subject to restrictions to protect water resources. However, the effectiveness of its actions relies on real coordination with other policies that affect it, which does not happen in practice.

The most important and widespread areas of land use restrictions in Brazil are conservation units (or protected areas). According to the National System of Nature Conservation Units, they are territorial spaces and their environmental resources, including jurisdictional waters, with relevant natural characteristics, legally established by the public authority, with conservation objectives, and defined limits, under a special administration regime, to which adequate protection guarantees apply (Brasil, 2000).

Protected areas have a wide range of management aims and are governed by many different stakeholders (Dudley, 2008). In Brazilian law, there are twelve categories of protected areas, subdivided into two main groups, with different specific objectives, protection methods, and permitted uses (Brasil, 2000). These characteristics are summarized in Table 1.

**Table 1:** - Table summarizing the characteristics of Brazil’s types of protected area

Group	Aim	Category	IUCN category	Objectives	Property / domain
Strictly protected area	Maintenance of ecosystems free from changes caused by human interference, allowing only the indirect use of their natural attributes	Ecological Station (ES)	Ia	preservation of nature and carrying out of scientific research	Public
		Biological Reserve (BR / ReBio)	Ia	full preservation of the biota and other natural attributes existing within its limits, without direct human interference or environmental modifications, except for measures to recover altered ecosystems and management actions necessary to recover and preserve the natural balance, biological diversity, and natural ecological processes	Public
		Wildlife Refuge	Ib	protect natural environments where conditions are ensured for the existence or reproduction of species or communities of local flora and residents or migratory fauna	Private or public
		National Park (NP)	II	preservation of natural ecosystems of great ecological relevance and scenic beauty, enabling scientific research and the development of environmental education and interpretation activities, recreation in contact with nature, and ecological tourism.	Public
		Natural Monument (NM)	III	preserve rare, unique, or scenically beautiful natural sites	Private or public
Sustainable use area	Exploitation of the environment in a way that guarantees the sustainability of renewable environmental resources and ecological processes, maintaining biodiversity and other ecological attributes, in a socially fair and economically viable manner	Area of Relevant Ecological Interest (AREI)	III	maintain natural ecosystems of regional or local importance and regulate the admissible use of these areas, to make it compatible with the objectives of nature conservation.	Private or public
		Private Reserve of Natural Heritage (PRNH)	IV	conserve biological diversity	Private
		Environmental Protection Area (EPA)	V	protect biological diversity, regulate the occupation process, and ensure the sustainable use of natural resources	Private or public
		National Forest (NF / FloNa)	VI	sustainable multiple uses of forest resources and scientific research, with emphasis on methods for the sustainable exploitation of native forests	Public
		Extractive Reserve	VI	protect the livelihoods and culture of these populations, and ensure the sustainable use of the unit’s natural resources	Public, with use granted to traditional extractive populations
		Wildlife Reserve	VI	technical-scientific studies on the sustainable economic management of wildlife resources	Public
		Sustainable Development Reserve	VI	preserve nature and, at the same time, ensure the conditions and means necessary for the reproduction and improvement of the ways and quality of life and exploitation of natural resources of traditional populations, as well as value, conserve, and improve the knowledge and techniques of environmental management developed by these populations.	Public, with regulated use by traditional populations

Elaborated by the authors, based on Law 9985/2000 (Brasil, 2000).

As shown in Table 1, there is some correspondence between the Brazilian categorization of Conservation Units and the International Union for Conservation of Nature (IUCN) classification of Protected Areas Management Categories. Reclassification to the IUCN standard enables the method to be comparable and applicable across different jurisdictions and global governance contexts.

The IUCN protected area categories receive their Roman numeral in an ordered form, where the categories Ia and Ib are highly protected sites where few, if any, people are allowed to enter, through parks where the emphasis is on conservation, but visitors are welcome. While higher numbers present less restrictive approaches where conservation is integrated into the traditional (and sometimes not so traditional) human lifestyles or even takes place alongside limited sustainable resource extraction (Dudley, 2008).

## Methods

### Study Area

**Fig. 1:**
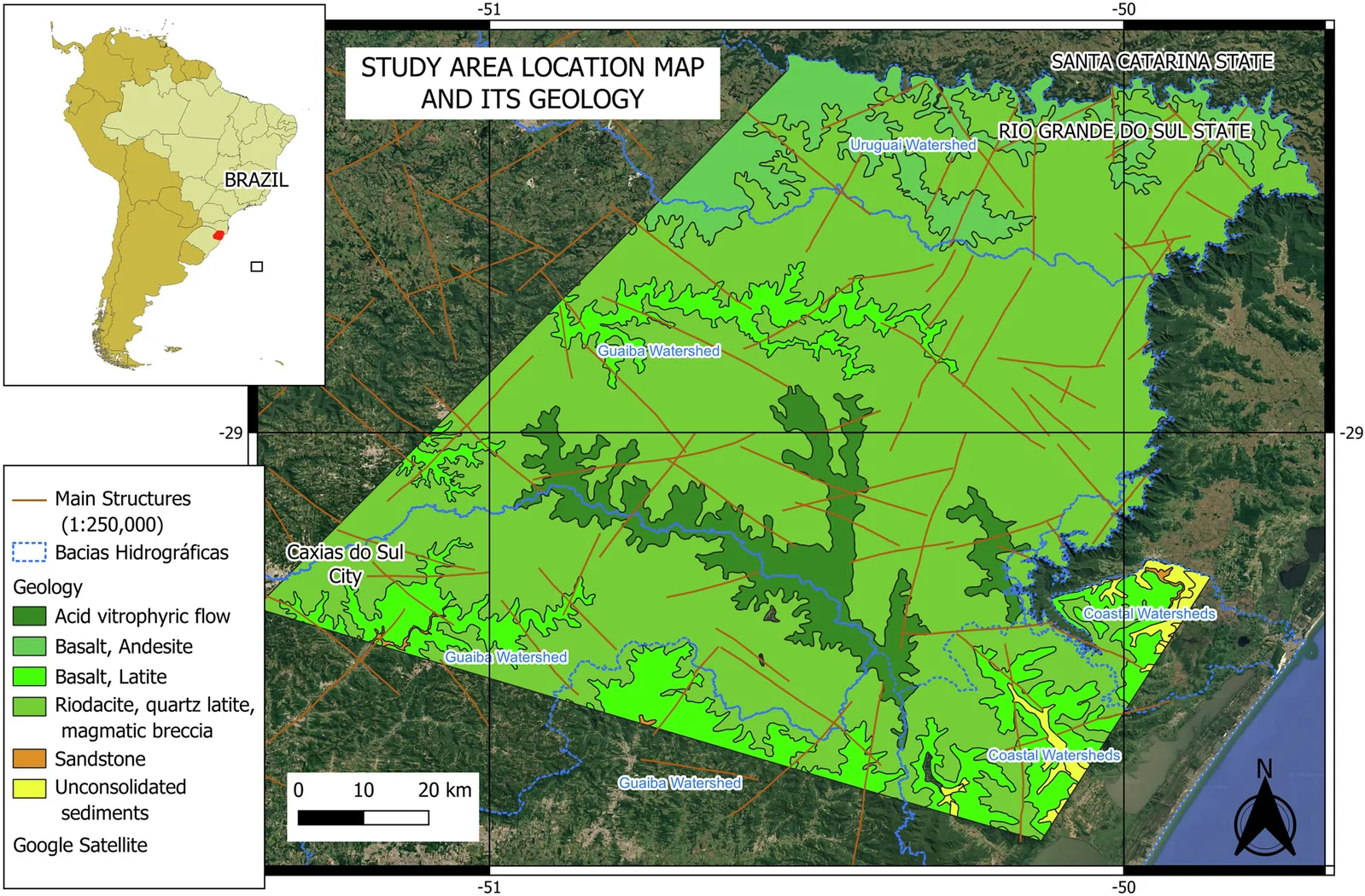
Study area location and geological map

The study area is situated in southern Brazil, in the northeast of the state of Rio Grande do Sul, between coordinates 49.6°W to 51.4° W and 28.4°S to 29.7° S (Fig. 1). Its boundaries are a dorsal fault system to the west and steep escarpments to the east and south. The north boundary is the Pelotas-Uruguay River, which marks the border of the states of Rio Grande do Sul and Santa Catarina.

The area primarily consists of a gently sloping plateau, inclined slightly to the west, composed of volcanic rocks from the Serra Geral Formation, within the geological framework of the Paraná Basin. This volcanic package is related to the opening of the South Atlantic and consists of basalts in most of the sequence and rhyolites and quartz latites in the upper portion (De Vargas et al., 2022), both of which are intensely fractured. The greater resistance to weathering and physical degradation of the rhyolites and quartz latites reinforces the process of escarpment regression due to falling blocks, creating a unique landscape on the eastern plateau border of the study area.

The terrain’s undulations are gentle, with an amplitude of up to 20 m. The high altitudes, predominantly over 800 m and reaching about 1400 m, combined with the location on the plateau’s edge near the coastal plain, contribute to regular and high rainfall (between 101 mm and 174 mm monthly) and average temperatures (between 11.4°C and 22.1°C) (Pereira et al., 2009).

The ecosystem has developed resilience to more extreme environmental conditions, consisting mainly of grassland, with intermittent ombrophile forest with *Araucaria Angustifolia* of the Atlantic Forest Biome. The weathering mantle is commonly thin (less than 2 m) and presents exudations (wetlands) alternated with dry zones (De Vargas et al., 2022). The grassland is traditionally used for extensive livestock farming, a low environmental impact activity. More recently, the regional economic development has been dominated by agriculture and tourism.

The drainage pattern is rectangular and strongly controlled by structural lines in several directions. The volcanic rock structures, fractures, and vesicular, amygdaloidal, and horizontal disjunction zones allow the exploitation of large volumes of groundwater at economically viable depths (IBGE, 2015). The Serra Geral Aquifer System is the most exploited in southern Brazil, and it constitutes an important or exclusive water supply for municipalities and rural producers.

This humid and temperate plateau comprises the headwaters for the three hydrographic regions of Rio Grande do Sul State: Uruguai (in the north), some coastal watersheds (to the east), and Guaíba (most of the area). The Guaíba hydrographic region has a large industrial and urban concentration, serving more than half of the state population. It has been affected by extreme events, notably the largest environmental disaster in Rio Grande do Sul’s history, which occurred in 2024, with various forms of environmental and human damage (Faria and Skamvetsakis, 2025). Trends show that some regions of the Guaíba hydrographic region may present high and low extremes of flow and precipitation, surpassing observed historical marks (Vieira et al., 2022).

**Fig. 2:**
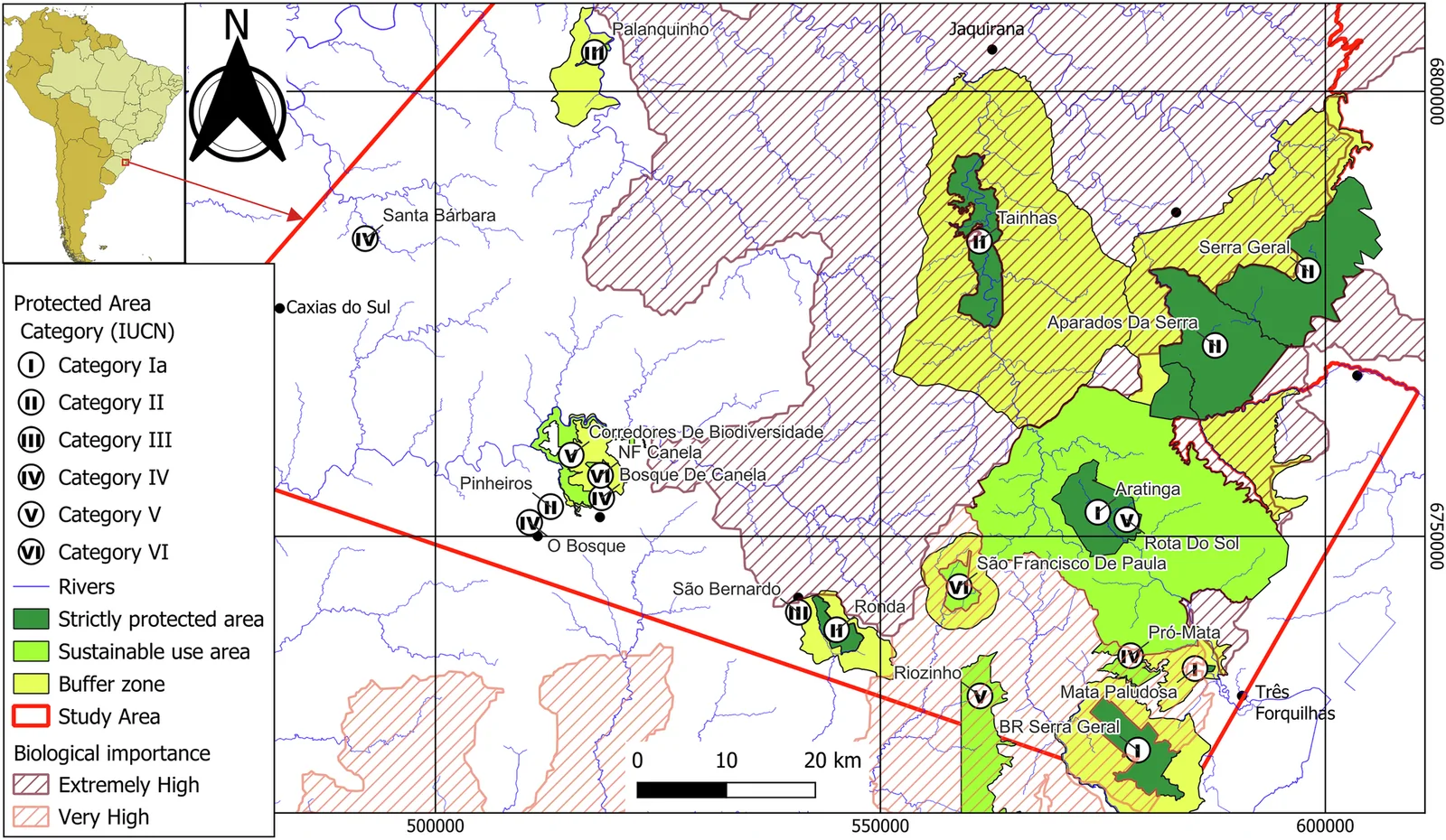
Location of the study area, highlighting the existing protected areas (UTM zona 22, Sirgas 2000, EPSG 31982)

**Fig. 3:**
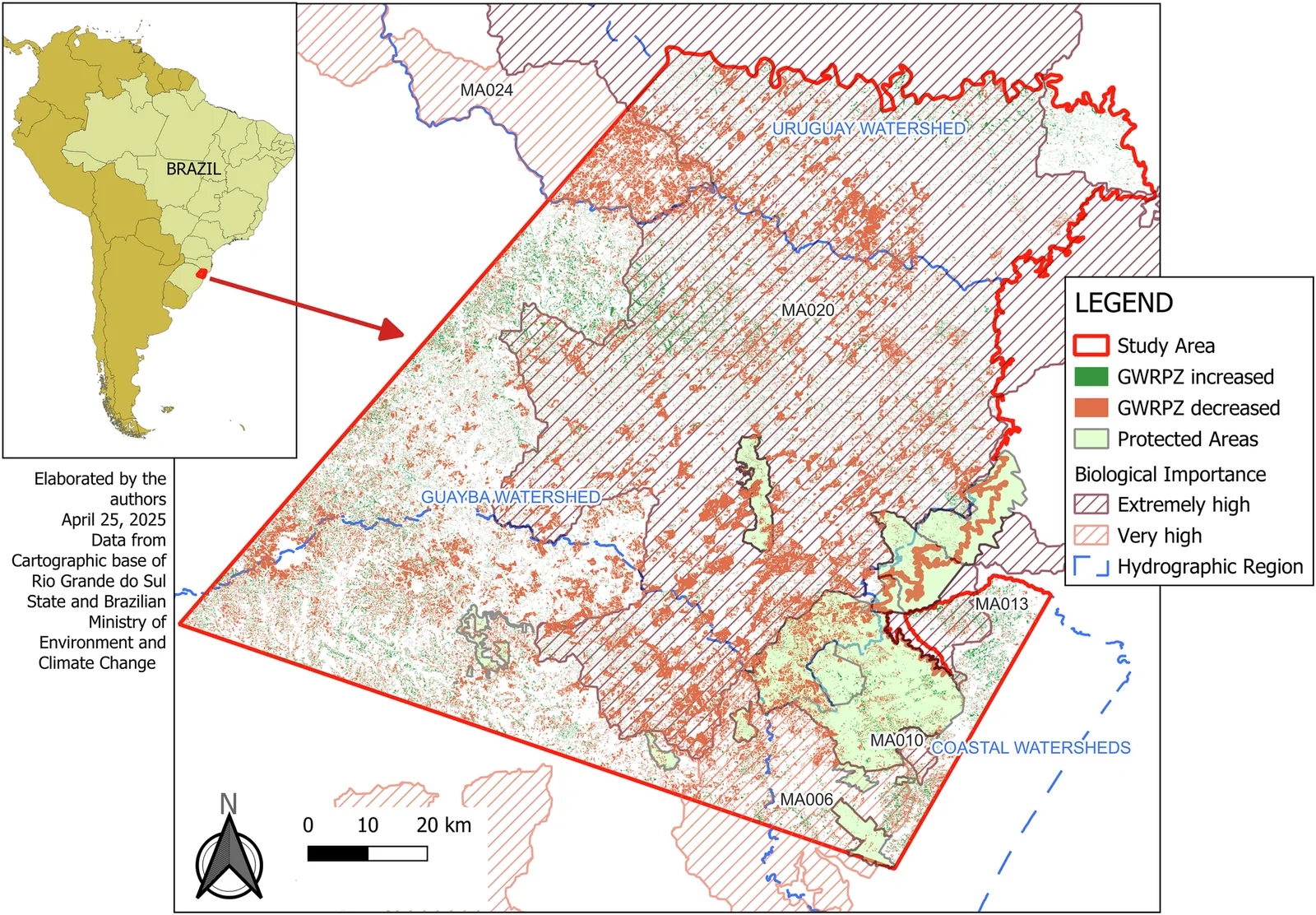
Groundwater recharge potential zone change map, with superposed territorial units of analysis

Of the total study area of 12,303 km^2^, 950 km^2^ (8%) is in protected areas, either strictly protected or sustainable use areas (Figs. 2 and 3). More than half (6398 km^2^, 52%) is classified as extremely high biological importance for conservation, for which the creation of protected areas is a high or extremely high priority (Brasil, 2023).

Another 138 km^2^ (1% of the study area) has very or extremely high importance priority, defined as the integrated and participatory management of protected areas, ecological corridors, and territories of traditional peoples and communities (Brasil, 2023). A further 810 km^2^ (7% of the study area) is considered of very high biological importance and of high priority for the recognition of Indigenous lands and the limitation or regularization of degrading activities (Brasil, 2023).

Being the source area for almost all the watersheds of an entire state and comprising the headwaters of densely populated valleys subject to flooding and inundations, in addition to totaling 68% of protected areas or areas of interest for protection, this study area has been considered ideal for an analysis of the efficacy of territorial planning for water security.

### Data Sources

The data used in this study include physical geographic data and documentary data, as shown in Table 2.

**Table 2 -:** Spatial data sources and scales

Spatial Dataset	Source / Reference	Original Scale or Spatial Resolution
Digital Elevation Model (DEM)	Copernicus Global DEM (Sinergise European Space Agency, n.d.)	30 m
Cartographic Base (RS)	State Secretariat for the Environment (Rio Grande do Sul, 2018)	1:25,000
Land Use and Land Cover (LULC)	MapBiomas Collection 8.0, Level 1 (MapBiomas, 2023)	30 meters
Annual Precipitation (Normals)	National Institute of Meteorology (INMET, 2025)	Point data (3 inside study area), interpolated via IDW
Geology, Soils, and Geomorphology	Geological Survey of Brazil (SGB, 2024a)	1:50,000
Well Data	Groundwater Information System of Geological Survey of Brazil (SGB, 2024b)	Point data (351 wells in the area)
Structural Lineaments	Manual vectorization from hillshade images	1:50,000
Protected and Priority Areas for conservation	Ministry of the Environment and Climate Change (Brazil, 2023)	Official vector dataset

While high-resolution data (such as the 1:25,000 cartographic base) were prioritized, some thematic layers, such as geology and soil maps, were only available at a 1:50,000 scale. To ensure spatial compatibility within the GIS-based Multi-Criteria Decision Analysis (MCDA), all layers were processed at a consistent 30 m resolution, matching the Copernicus Global DEM and MapBiomas datasets.

### Groundwater Recharge Potential Zone Changes

The hierarchical weights and the hydrological rationale used in this study were established and validated in a recently published foundational paper by the same authors (Wiederkehr et al., 2025), which specifically focused on the impacts of land cover and climatic changes on the groundwater recharge of this volcanic plateau.

The GWRPZ map was elaborated using the Analytic Hierarchy Process (AHP), with the nine most influential criteria identified based on an extensive literature review of 98 studies regarding the Serra Geral Aquifer System. Lineament density was assigned the highest weight (0.347) because, in fractured volcanic aquifers, structural discontinuities are the primary pathways for vertical water infiltration and secondary porosity. Geology (0.169) and slope (0.160) were prioritized because they govern the balance between surface runoff and infiltration, especially considering the high slope variations at the plateau’s edge. The other parameters used were LULC (0.137), geomorphology (0.059), soil type (0.047), soil depth (0.036), drainage density (0.036), and annual rainfall (0.017). Factors such as soil type and depth were weighted based on their water storage capacity, which limits the amount of water reaching the fractured substrate.

The resulting consistency ratio was 0.056. Map removal and pairwise sensitivity analyses were also conducted to ensure the reliability of the weights. The accuracy of the resulting GWRPZ maps was validated using a dataset of 351 wells from the SIAGAS database. The results show that 70.3% of these wells (247 out of 351) are in areas identified by our model as having “High” or “Good” recharge potential. Significantly, no wells were found in areas classified as “Low” potential, which confirms the model’s accuracy in identifying viable aquifers. The resulting maps were also cross-validated using high-resolution Google Earth imagery to verify whether high-potential zones matched physical features such as major lineament intersections, water bodies, and areas with dense natural vegetation.

All thematic maps were labeled separately according to the calculated weights and integrated using GIS functions to produce the recharge potential map. Elevation, slope, geology, soil, geomorphology, lineament density, and drainage density are long-term parameters, but LULC and the climate have undergone notable shifts recently and have impacted the GWRPZs. The parameters that changed over the past decades, precipitation and LULC, were obtained and analyzed separately for two distinct periods, the 1980s and 2020 s, to understand how these changes impacted the study area’s GWRPZs over time.

These two maps were compared in GIS using change detection analysis, which highlighted the areas where GWRPZ loss or gain occurred. The change detection was performed using the Raster Calculator method within a GIS environment. This allowed for a cell-by-cell comparison to identify net gains and losses in potential between 1985 and 2022.

### Analysis of the Protected Areas

Within the study area, there are nineteen protected areas (Fig. 2, Table 3). Nine of them are strictly protected areas, and ten are sustainable use. They were reclassified according to the protected area management categories proposed by the IUCN (Dudley, 2008) to facilitate the comprehension and comparison within different countries and regions, according to the correspondence established in Table 1.

**Table 3 -:** GWRPZ changes within the study area’s LULC-regulated areas

	Total area (km^2^)	GWRPZ increase (km^2^)	GWRPZ decrease (km^2^)	GWRPZ net change (km^2^)	GWRPZ increase (%)	GWRPZ decrease (%)	GWRPZ net change (%)	IUCN category mean normalized net change (%)
BR MP	2.7205	0.1981	0.1368	0.0614	7.2824	5.0269	2.2555	I (-1.39%)
BR SG	48.4890	0.2761	0.9637	-0.6875	0.5695	1.9874	-1.4179	
ES Ara	60.4140	2.1070	3.0319	-0.9249	3.4876	5.0185	-1.5309	
NP SG	86.6860	1.5245	3.2538	-1.7293	1.7586	3.7535	-1.9949	II (-6.04%)
NP Apa	75.6620	1.2973	3.1128	-1.8155	1.7146	4.1141	-2.3995	
NP Pin	1.1664	0.0802	0.1097	-0.0295	6.8721	9.4024	-2.5303	
NP Ron	14.4930	0.2472	1.8477	-1.6005	1.7057	12.7489	-11.0433	
NP Tai	66.5938	1.6580	11.2660	-9.6080	2.4897	16.9175	-14.4278	
NM Pal	2.5120	0.1967	0.0640	0.1327	7.8304	2.5478	5.2826	III ( + 4.99%)
AREI SB	0.1814	0.0045	0.0027	0.0018	2.4807	1.4884	0.9923	
PRNH SB	0.0200	0.0019	0.0009	0.0010	9.6635	4.4338	5.2296	IV (-0.95%)
PRNH OB	0.0679	0.0000	0.0000	0.0000	0.0000	0.0000	0.0000	
PRNH PM	23.9776	0.5034	0.7287	-0.2253	2.0996	3.0391	-0.9395	
PRNH BC	0.0581617	0	0.00465	-0.0047	0.0000	7.9950	-7.9950	
EPA CC	26.9016	0.5503	2.1325	-1.5822	2.0456	7.9271	-5.8814	V (-13.81%)
EPA Rio	30.3794	1.3660	3.8478	-2.4818	4.4965	12.6658	-8.1694	
EPA RS	486.6420	16.3160	87.3584	-71.0424	3.3528	17.9513	-14.5985	
NF Can	5.6350	0.2202	0.9340	-0.7138	3.9077	16.5750	-12.6673	VI (-18.20%)
NF SFP	16.1560	0.2085	3.4616	-3.2531	1.2905	21.4261	-20.1356	
NM Pal Buffer	48.0544	3.5706	4.1724	-0.6018	7.4303	8.6826	-1.2523	BZ (-16.52%)
BR SG Buffer	90.5366	4.4450	6.9798	-2.5348	4.9096	7.7094	-2.7998	
NP SG/ApaBuffer	349.0709	12.2167	49.9562	-37.7395	3.4998	14.3112	-10.8114	
NF Can Buffer	47.0650	1.6287	8.2788	-6.6501	3.4605	17.5901	-14.1296	
MP Ron Buffer	47.4560	0.8246	8.1471	-7.3225	1.7376	17.1677	-15.4301	
NP Tai Buffer	614.1330	11.0894	147.7680	-136.6786	1.8057	24.0612	-22.2555	
NFSFP Buffer	53.1097	1.0237	15.8607	-14.8370	1.9275	29.8640	-27.9365	
Other areas	10031.60	413.66	1617.90	-1204.2380	4.1236	16.1280	-12.0044	OUT (-12.00%)
Study Area	12303.80	479.70	1994.63	-1514.9330	3.8988	16.2115	**-12.3127**	ALL (-12.31%)

**Note:** Percent changes are normalized by each protected area’s total area. Category values represent the mean normalized net change (%) across protected areas within each IUCN category.

In Brazilian law, the surroundings of a conservation unit are subject to specific rules and restrictions for human activities to minimize negative impacts on the protected area. This is called a buffer zone, and it is mandatory for all types of conservation units, except environmental protection areas and private natural heritage reserves (Brasil, 2000).

Thus, the study area includes eight buffer zones. One of them, the buffer zone of the Aratinga Ecological Station (ES Ara), overlaps with the Rota do Sol Environmental Protection Area (EPA RS). Because São Francisco de Paula National Forest is the only protected area without an official buffer zone, its buffer was defined as a 2000 m zone, following the recommendation of the National Environmental Council (Conselho Nacional do Meio Ambiente, 2010).

The contours of these LULC-regulated areas were used to clip the vector layer of GWRPZ changes. Each new layer was analyzed using the field calculator of the attribute table to calculate the total area and the GWRPZ area loss and gain. The data were tabulated to calculate the net loss (in km^2^ and %) of each conservation unit, each category of conservation unit, and each main group of use restriction.

## **Results**

### Overall Spatial-temporal Dynamics and LULC Drivers

The resulting GWRPZ change map showed areas where recharge potential improved and others where recharge potential was reduced between 1985 and 2022 (Fig. 3). Overall, the study area (12303.8 km^2^) gained 479.7 km^2^ (3.9%) of GWRPZ and lost 1514.9 km^2^ (-16.2%). Hence, the net loss of GWRPZ was 12.3%, despite a slight increase in rainfall.

**Fig. 4:**
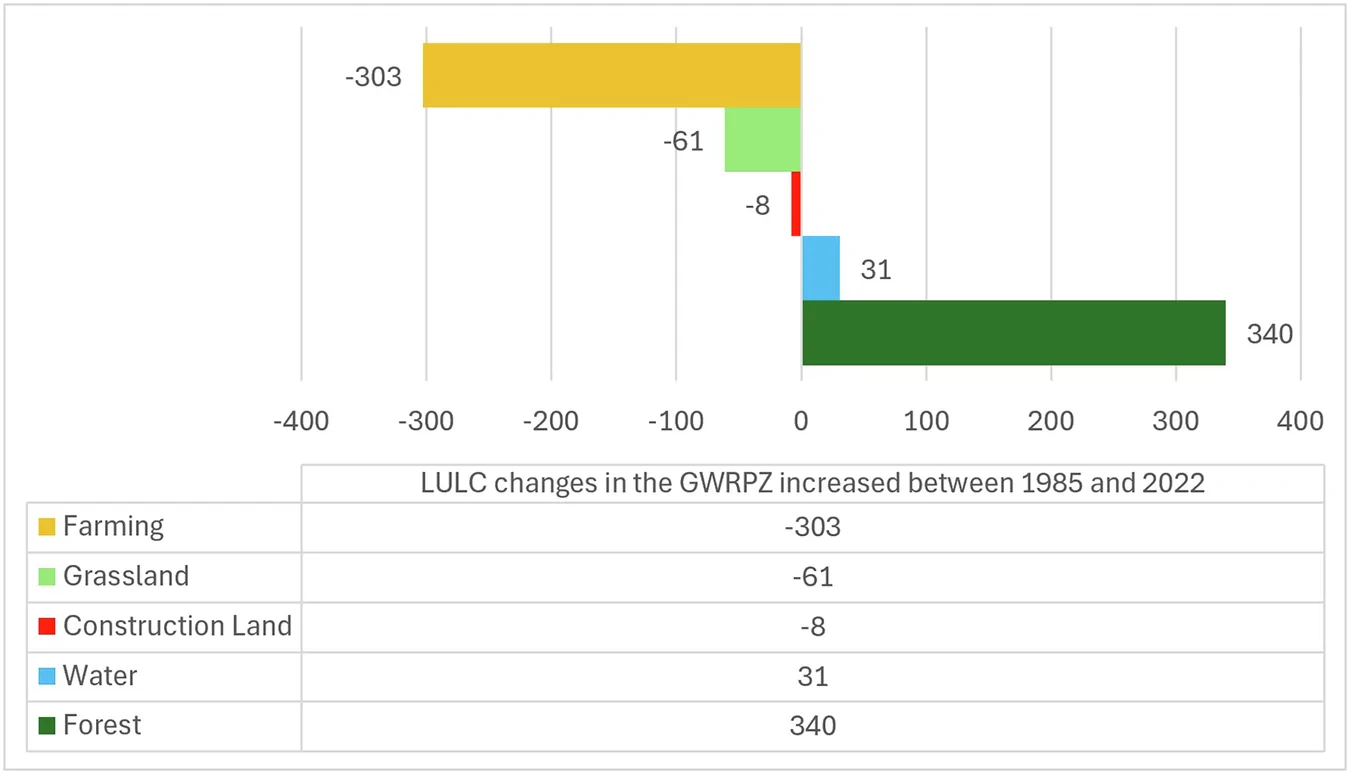
LULC changes within the areas of GWRPZ decrease between 1985 and 2022 in the study area, showing that the main reason for GWRPZ loss was conversion to farmland

**Fig. 5:**
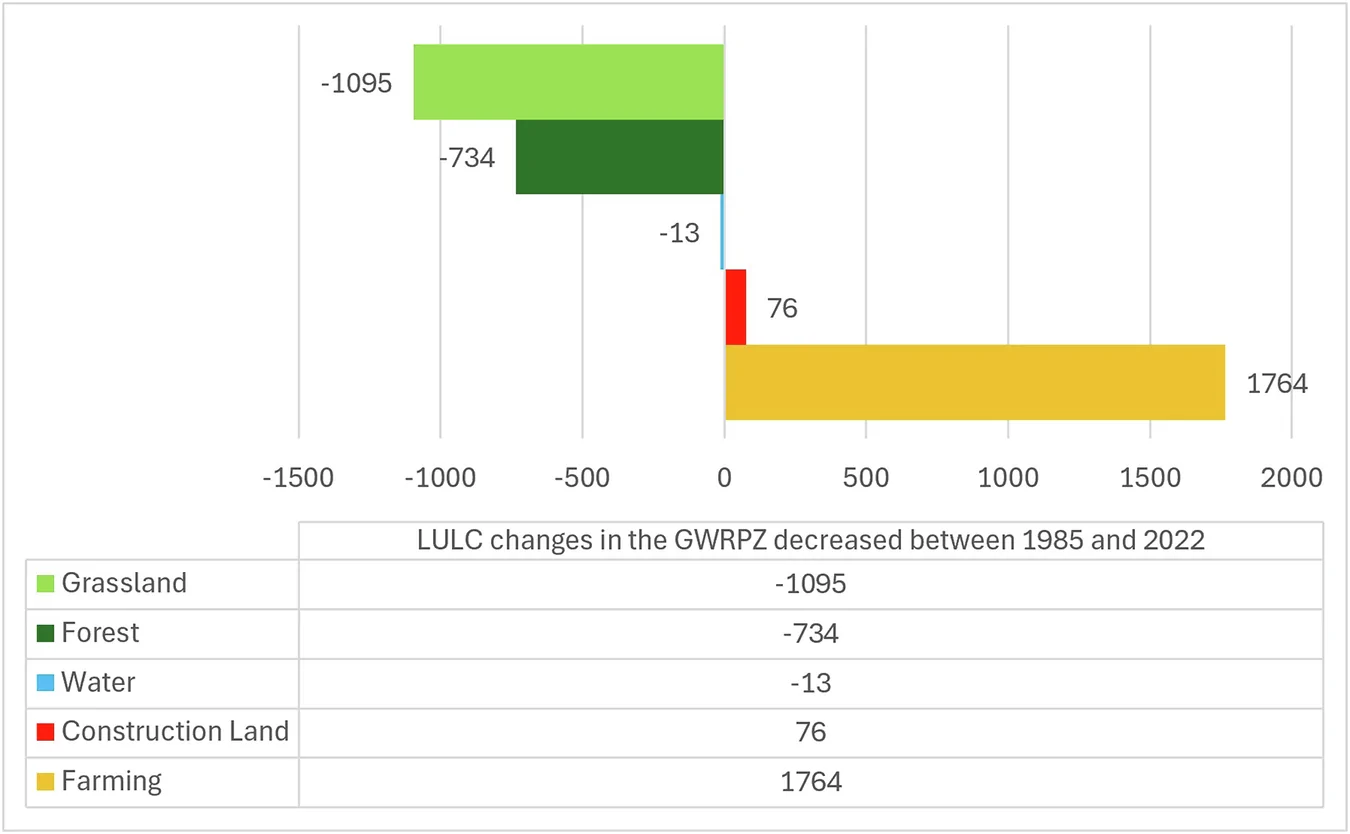
LULC changes within the areas of GWRPZ increase between 1985 and 2022 in the study area, showing that the main reason for the improvement of recharge zones was forest recovery

The analysis of the LULC changes in the areas of GWRPZ reduction showed that the main pressure affecting the infiltration rates was the widespread conversion of forests and grassland into farmland, followed by punctual urban growth (Fig. 4). Places where there was a GWRPZ gain mainly showed forest recovery, but there were also new bodies of water. That indicates that the main resistance factors for GWRPZ were the forests, wetlands, and small dams (Fig. 5).

### Effectiveness of Regulated Areas

The analysis of individual protected areas indicates that the units with the smallest area (less than 5 km^2^) tended to present the best net GWRPZ change results. The only five conservation units that present a net GWRPZ gain or insignificant change between 1985 and 2022 are in this class. In other words, protected area management categories III and IV, the study area’s smallest conservation units, presented the best results (Table 3). That may indicate that small conservation units are easier to protect. It is important to consider, however, the possible influence of the scale of work in that context.

Among the conservation units with more than 5 km^2^ within the study area, the results showed a direct correspondence to the degree of environmental modification predicted by the IUCN for categories I (-1.39%), II (-6.04%), and V (-13.81%). All categories showed a net loss of GWRPZs, but this loss is small in the categories with more restricted uses, increasing progressively with the category numbers (Table 3).

However, an inversion was observed in this pattern in the more permissive areas. It was expected that category VI, sustainable use protected areas, would suffer fewer human interventions than the buffer zones, and those were expected to have been less affected than the areas without any special LULC regulation. Of the regulated areas, GWRPZ loss was smaller (12%) than in sustainable use categories V (13.8%) and VI (18.2%). There was a net GWRPZ loss of 16.52% in the buffer zones (Table 3). This demonstrates the pressure on the protected areas in the region.

#### Strictly Protected Areas

Strictly protected areas generally maintained GWRPZ better than less restrictive categories, although performance varied according to management effectiveness, land regularization, and surrounding pressures. The best results occurred in Biological Reserve Mata Paludosa (BR MP), which showed a net GWRPZ increase of 2%, and in Biological Reserve Serra Geral (BR SG) and Ecological Station Aratinga (ES Ara), where losses were limited to 1.4% and 1.5%, respectively. In these areas, forest recovery and strict limits on infrastructure and land-use change helped offset or minimize negative impacts, even where parts of the territories still require regularization.

The national parks Serra Geral and Aparados da Serra also presented relatively small GWRPZ losses, of 2% and 2.4%, respectively. The main pressure in these units was the conversion of native grasslands to agriculture. Pinheiros National Park, created more recently, lost 2.5% of its GWRPZ, mainly due to deforestation, partly compensated by the construction of a dam.

The weakest results among strictly protected areas were observed in Ronda National Park and Tainhas National Park. Ronda lost 11% of its GWRPZ, associated with exotic forestry, farming, and wood removal, while Tainhas lost 14.4%, reflecting land regularization problems and conflicting activities such as exotic invasive forestry and grassland conversion.

#### Sustainable Use Areas

Sustainable use areas showed mixed results, with performance strongly related to size, management intensity, and surrounding land-use pressures. The best outcomes occurred in small, well-managed units such as Natural Monument Palanquinho, PRNH Santa Bárbara, and AREI São Bernardo, where forest recovery, reduced fragmentation, ecotourism, or limited recreational use contributed to stable or increased GWRPZ. PRNH O Bosque remained unchanged because it is fully forested, while PRNH Pró-Mata showed only minor changes.

In contrast, larger or more permissive sustainable use areas generally experienced greater GWRPZ losses. PRNH Bosque de Canela was affected by deforestation and urban expansion, while the Environmental Protection Areas showed losses linked mainly to deforestation, urban growth, and conversion of grasslands to farmland. EPA Rota do Sol, despite its role as a buffer and ecological corridor, lost 14.6% of its GWRPZ, including within zones intended for water and wildlife conservation.

The weakest results were observed in the national forests, where sustainable wood use and associated LULC changes are likely to increase GWRPZ variation. NF Canela lost 12.7% of its GWRPZ, while NF São Francisco de Paula lost 20.1%.

#### Buffer Zones

Buffer zones showed heterogeneous but generally negative GWRPZ trends, indicating strong land-use pressure around protected areas. The lowest losses occurred in the BR Serra Geral and NM Palanquinho buffer zones, with net reductions of 2.8% and 1.25%, respectively. In both cases, some conversion of natural formations to farmland occurred, but forest recovery, reverse land-use changes, or water accumulation associated with a small hydroelectric plant helped reduce the net impact.

Other buffer zones showed losses near or above the study-area average, mainly due to the conversion of grasslands and forest patches into farmland, as well as proximity to urban areas and roads. The buffer zone shared by Aparados da Serra and Serra Geral National Parks lost 10.8% of its GWRPZ, while the Ronda National Park buffer zone lost 15.4%.

The most critical losses occurred around Tainhas National Park and the national forests. The Tainhas buffer zone lost 22.2% of its GWRPZ, while the NF São Francisco de Paula buffer zone had the largest reduction in the study, reaching 27.9%. Overall, these results show that buffer zones are often more pressured than the protected areas they surround, revealing weaknesses in regulation, surveillance, and land-use control.

### Synthesis

When analyzing the two main groups of protected areas in comparison with unregulated territories, the spatial mapping highlights the role of strict conservation in maintaining groundwater recharge potential zones. Here, the percentage of GWRPZ changes was calculated using the total area of the conservation units and the net loss within the group. The higher the GWRPZ maintenance, the higher the degree of protection of the area, as shown below:

- Strictly Protected: -2.68%
- Sustainable Use: -7.24%
- Protected area buffer zone: -13.52%
- Out of regulated areas: -12.00%

Outside the regulated areas, however, it is noteworthy that the conservation units’ buffer zones have greater potential recharge area loss than areas without specific regulation. This demonstrates the pressure on protected areas and reflects the difficulties in regulating and surveilling these areas.

### Another Territorial Analysis

The headwaters of the Guaíba Hydrographic Region, totaling 8,626.25 km^2^, lost 331.6 km^2^ and gained 353.7 km^2^ of GWRPZ. The net GWRPZ loss in this basin within the study area was 13.86%, above the study area average.

The headwaters of the Uruguai River Hydrographic Region, in the northern study area, comprises an area of 2322 km^2^. Between 1985 and 2022 its GWRPZ increased by 58.8 km^2^ and decreased by 331.6 km^2^, resulting in a change of -11.7%, below the study area average. The LULC alterations were concentrated in the western portion of this basin in the study area.

Among the hydrographic regions, the best GWRPZ maintenance was found in the coastal watersheds, in the eastern study area. Of a total area of 1354 km^2^, their GWRPZ area increased by 67.2 km^2^ and decreased by 113.3 km^2^, for a change of just -3.4%.

Three priority conservation areas presented GWRPZ loss above the study area average. The MA-24 unit showed the worst result in the study, with recharge potential reduced by 40% within its territory. The MA-20 unit, the largest and most important conservation area in the study, had a GWRPZ loss of 13.8%, near the study area average.

## Discussion

This study contributes to international scholarship by moving beyond static groundwater recharge potential mapping and examining how recharge potential zones changed over nearly four decades under different land-use protection regimes. While GIS-based GWRPZ mapping has been widely applied in hydrogeological studies, fewer studies have explicitly connected spatial-temporal recharge potential dynamics with protected-area categories, buffer zones, and territorial planning policies. In this sense, the study provides a transferable framework for assessing how land-use governance may be associated with the maintenance or loss of recharge potential in fractured volcanic plateau headwaters. This contribution is relevant to other volcanic and crystalline aquifer regions worldwide, where recharge is spatially heterogeneous, land-use change is increasing, and groundwater management depends on integrating hydrogeological evidence with territorial planning.

This study connects biodiversity priority areas with priority areas for water supply and flow regulation ecosystem services. As demonstrated by Egoh et al. (2011), integrating ecosystem services and biodiversity into a single spatial planning framework can generate combined benefits, linking biodiversity conservation with the maintenance of essential ecosystem functions.

For adequate environmental management, it is essential to recognize the main pressures and resistances on the resource to be protected. Here, it was demonstrated that the main pressure acting in the study area was conversion to farmland, although urbanization also played an important role. Farming has been growing rapidly since 2000, driven mainly by seasonal soybean crops (MapBiomas, 2023).

In the study area, the pressure on grassland is notable. Nowadays, these high-altitude grasslands are protected by Brazilian laws, but they are threatened by a bill that would turn non-forest vegetation into consolidated rural areas subject to land use conversion, provided they were used for agrosilvopastoral activities before 2008 (Brasil, 2019). But grasslands and savannas in Brazil have historically been used for cattle grazing, in many cases without significant changes in the structure and functioning of the ecosystem or in the species diversity. Analyses suggest that the bill, if approved, has the potential to allow the conversion of vast areas of non-forest vegetation across all Brazilian biomes to other uses.

The main resistances identified in the study area were forest recovery and the presence of water bodies. It was observed that small protected areas (less than 5 km^2^) tended to exhibit forest recovery that improved the GWRPZ. This pattern may reflect the lower monitoring and management complexity of small protected areas. Meanwhile, larger areas with less restrictive rules tended to present recharge area loss above the study area average. However, size is not a strict rule, since the areas studied with above-average GWRPZ loss varied from nearly five to more than 600 km^2^ in size.

It is important to consider, however, that this study did not distinguish natural forests from afforestation, and some research has demonstrated that the replacement of grasslands or pasture for eucalyptus (Mattos et al., 2019), or pine (Adane et al., 2018), or even forests (Amano and Iwasaki, 2020) can reduce groundwater recharge.

With some exceptions, the results indicate a positive relationship between the degree of use restriction and the preservation of groundwater recharge potential characteristics. However, it is noteworthy that the buffer zones of protected areas have been suffering more pressure than areas without any special use regulation. This may be due to the location of these areas, pressure from economic activities, or the lack of effectiveness of protective measures.

In terms of the hydrographic regions, the one that showed the greatest GWRPZ loss was precisely the one that witnessed the greatest disaster in Rio Grande do Sul, in May 2024. Floods in various watersheds of the Guaíba Hydrographic Region are common and recurring. While spatial management of headwater recharge potential zones provides a critical baseline for landscape protection, evaluating whether recharge preservation directly reduces downstream flood peaks requires future dynamic hydrological and hydrograph modeling.

By knowing the main pressures and resistances, and the areas more vulnerable to these pressures or more conserved by their resistances, it is possible to plan territorial management and promote sustainable development that would benefit the whole state, not just the study area. The limits of farmland expansion for sustainable development in terms of hydric insurance may be evaluated by simulations and modeling. The concept of environmental compensation is defined in the Constitution of the Federative Republic of Brazil and could be considered in future policy discussions involving municipalities that face development restrictions due to the protection of groundwater recharge potential areas. In this context, compensation mechanisms may help align local development constraints with broader territorial planning goals aimed at maintaining recharge-related ecosystem functions.

According to Wang et al. (2024), the integrated consideration of pressure and resistance can help prevent unnecessary and excessive governance of protected areas, and determine where to allocate resources and ensure appropriate investments in conservation. Although this study demonstrates the efficacy of LULC restrictions in maintaining groundwater recharge potential, the intention is not to convert the entire plateau into strictly protected areas.

It is necessary to carry out studies from the perspective of water security to aid the decision-making process for creating new conservation units in the region and adopting policies that promote the sustainable development of the area, while valuing the historic-cultural character and tourism potential of the study area. The empowerment of local people and equitable benefit-sharing increases the likelihood of effective conservation (Das, 2024).

On fractured aquifers, where preferential flows dominate through rock fractures, the major recharge mechanism is focused recharge, rather than diffuse recharge (Yenehun et al., 2022). Focused recharge tends to be localized and more spatially variable (Kebede et al., 2024), as observed by Yenehun et al. (2022) across the Ethiopian volcanic rock aquifers. Understanding future groundwater availability and related surface flows under normal management is necessary in order to develop policy adjustments that will enhance water sustainability (Sishodia et al., 2018).

Another similar volcanic plateau around the world, although of different ages, is facing groundwater depletion, which tends to get worse with LULC changes and climatic changes. As examples can be cited the Deccan Plateau in India (Sishodia et al., 2018), the Columbia Plateau in the United States of America (Asante-Sasu et al., 2025), the Ethiopian Plateau in Africa (Yenehun et al., 2022), among others. All of them present significant spatial heterogeneity that needs to be considered in groundwater studies and management.

India is the largest groundwater user in the world, contributing to more than 25% of the global annual withdrawals. The crystalline aquifer region of central and south India has been experiencing depletion and environmental issues, which tend to get worse due to climatic and societal changes coupled with limited rainfall and adaptation capacity (Sishodia et al., 2018). The basalt bedrock posed serious constraints on groundwater storage, which contributed to regular agricultural droughts.

The Columbia Plateau is mainly occupied by cropland. The groundwater withdrawals are mostly for irrigation purposes due to a semiarid climate (Asante-Sasu et al., 2025). The arid grasslands of this plateau have historically been used for grazing, as the study area of this work, but have been strongly converted into agricultural lands. Today, water quality and quantity are a particular concern in this region.

In the East African Rift Valley, water availability in the rift valley relies heavily on the discharge from the highlands. So, Yifru et al. (2021) studied the effect of LULC and climate change on water yield and groundwater recharge on the bounding volcanic plateaus and identified a strong relationship between the presence of forest and shrubland and the spatial distribution of recharge.

In north-western Namibia, a flat-lying volcanic rock sequence of the Etendeka Formation crops out. Extensive dolerite sills and dyke swarms are related to the volcanic rocks, which have formed in connection with the continental breakup of Gondwanaland (Christelis et al., 2001), as well as the Serra Geral Aquifer System. Situated in an arid climate, the Etendeka Table Mountain landscape is dependent on very limited groundwater supplies. The groundwater potential of these volcanic rocks is still little known; the stored reserves are limited, and recharge is low and highly dependent on the infrequent rainfall (Christelis et al., 2001).

The pressure on the grasslands is not exclusive to the study area. During the past century, grasslands have decreased globally mainly because of conversion to arable land to produce animal feed crops (Bengtsson et al., 2019; Parr et al., 2014). This non-forest vegetation plays an important role in several ecosystem services highly demanded by society, such as water supply and flow regulation, carbon storage, erosion control, cultural values, pollination, and biological control of agricultural pests (Bengtsson et al., 2019).

Grassland degradation is insidious and less visible than degradation in forested biomes (Parr et al., 2014). Grasslands are much more at risk of transformation into other land uses than forests, and where they are protected, management is often inadequate because it excludes necessary disturbances (Overbeck et al., 2018). Bengtsson et al. (2019) showed that the societal demand for ecosystem services is often matched by supply from natural grasslands, suggesting that policies could focus on improving the management of existing grasslands and mitigating their decrease and degradation.

To reduce economic activity pressure, financial compensation for eventual restrictions on land use can be offered from public resources to stimulate the sustainable use and protection of these plateau areas. However, the current demand for grassland climate regulation appears low to null (Bengtsson et al., 2019). According to Parr et al. (2014), it is important to thoroughly evaluate the results of policies geared towards maximizing forest cover for carbon, especially when aboveground carbon is emphasized.

### Limitations

A key conceptual limitation of this study is that the AHP-GIS framework delineates spatial potential zones for recharge based on static and dynamic multi-criteria indicators, rather than physically modeling subsurface water flux, streamflow hydrographs, or downstream flood peaks. While high recharge potential zones theoretically support infiltration and baseflow maintenance, direct quantification of flood peak reduction or drought mitigation requires empirical streamflow data and numerical hydrological modeling, which were beyond the scope of this spatial-temporal assessment.

This work used LULC Data Level 1, which does not distinguish different types of forest, herbaceous vegetation, or cultures, that may have different effects on groundwater recharge. For example, the spatial variability and spectral similarity between grassland and pasture are challenging to separate, even using hyperspectral images (Souza et al., 2020).

The study area is relatively large for work at a scale of 1:25000, as proposed, and some data were available only at a scale of 1:50000, affecting the spatial resolution of the GWRPZ map. Although the AHP-GIS approach used to generate the GWRPZ map was supported by validation and sensitivity analysis, uncertainties remain. The well-based validation supports the spatial plausibility of the recharge potential map, but it does not provide direct measurements of recharge rates or temporal changes in groundwater storage.

This map was clipped by protected-area shapefiles, and some of them are so small in relation to the working scale that the analysis may be compromised. Therefore, detailing strategic areas is important for future studies.

Finally, because few studies have examined the relationship between groundwater recharge potential zones and protected areas, the comparability of our results remains limited. In this context, this study provides a useful spatial-temporal benchmark for assessing how land-use and land-cover policies are associated with the maintenance or loss of recharge potential. The results can support spatial planning related to groundwater recharge potential and provide a basis for future assessments of water-related ecosystem services.

## Conclusions

This study assessed how territorial planning policies influence groundwater recharge potential zones (GWRPZs) in a fractured volcanic plateau in southern Brazil between 1985 and 2022. The validated GIS-based model, supported by well data and sensitivity analyses, provides an evidence base for linking land-use regulation, ecosystem conservation, and water security. The main conclusions are organized as follows:

1. **Groundwater recharge potential declined despite favorable rainfall conditions**. Between 1985 and 2022, the study area showed a net GWRPZ loss of 12.31%, even with a slight increase in precipitation. This indicates that land-use and land-cover change, rather than climate alone, was the main factor reducing recharge potential. The conversion of grasslands and forests to seasonal farming was the dominant pressure, while forest recovery and the presence of water bodies were associated with recharge gains.
2. **Territorial protection improves the maintenance of recharge functions**. Areas with stricter land-use restrictions preserved GWRPZs more effectively than less regulated territories. Strictly protected areas showed substantially lower net loss than unregulated areas (-2.68% versus -12.00%), demonstrating that conservation policies can inform protection of recharge-sensitive areas and provide a spatial basis for future hydrological and risk assessments. However, the high losses observed in buffer zones reveal that legal designation alone is insufficient when regulation, surveillance, and land-use control are weak.
3. **Grassland conversion is strongly associated with recharge potential loss**. The spatial-temporal analysis indicates that, in the studied fractured plateau headwaters, areas where high-altitude grasslands were converted to seasonal agriculture were among the main contributors to GWRPZ reduction between 1985 and 2022. Although grasslands are often less emphasized in conservation policies than forests, these results suggest that maintaining native non-forest formations can help preserve spatial recharge potential in this specific hydrogeological context.
4. **GWRPZ mapping can support management priorities**. The spatial identification of recharge loss and gain areas allows managers to locate priority zones for conservation, restoration, and land-use restriction. These maps can help define where agricultural expansion should be limited, where forest or grassland recovery should be encouraged, and where buffer zones require stronger enforcement. They also provide a spatial basis for future watershed planning and hydrological modeling by identifying upstream areas where changes in recharge potential may affect water storage and availability, while the effects on downstream flood peaks should be evaluated through specific hydrological simulations.
5. **The results provide guidance for public policy**. Public policies should integrate groundwater recharge criteria into territorial planning, protected-area management plans, ecological-economic zoning, watershed plans, and environmental licensing procedures. Policy instruments should prioritize the conservation of recharge zones, strengthen governance in buffer zones, regulate conversion of grasslands and forests to intensive agriculture, and consider compensation mechanisms for municipalities or landowners that maintain land uses compatible with water security.
6. **The proposed framework is transferable to other headwater regions**. Although this study focused on a volcanic plateau with fractured aquifers, the methodological framework can be adapted to other regions where groundwater recharge is spatially heterogeneous and strongly influenced by land use. Its application requires adjustment to local hydrogeological, climatic, ecological, and socioeconomic conditions, especially in areas where water security depends on the protection of recharge zones rather than on infrastructure alone.

Overall, the findings show that scientifically grounded territorial planning can help reconcile biodiversity conservation, agricultural development, and the maintenance of groundwater recharge potential. For management and policy, the central implication is that recharge potential zones should be treated as important spatial planning units in headwater regions. Their protection can help preserve recharge-related landscape conditions and provide a spatial basis for future hydrological assessments, watershed planning, and more sustainable land-use decisions.
